# Parkin recruitment to impaired mitochondria for nonselective ubiquitylation is facilitated by MITOL

**DOI:** 10.1074/jbc.RA118.006302

**Published:** 2019-05-20

**Authors:** Fumika Koyano, Koji Yamano, Hidetaka Kosako, Keiji Tanaka, Noriyuki Matsuda

**Affiliations:** From the ‡ Ubiquitin Project and; ¶ Laboratory of Protein Metabolism, Tokyo Metropolitan Institute of Medical Science, 2-1-6 Kamikitazawa, Setagaya, Tokyo 156-8506, Japan and; the § Division of Cell Signaling, Fujii Memorial Institute of Medical Sciences, Tokushima University, 3-18-15 Kuramoto-cho, Tokushima 770-8503, Japan

**Keywords:** parkin, Parkinson disease, mitophagy, ubiquitin, ubiquitin ligase

## Abstract

*PINK1* (*PARK6*) and *PARKIN* (*PARK2*) are causal genes of recessive familial Parkinson's disease. Parkin is a ubiquitin ligase E3 that conjugates ubiquitin to impaired mitochondrial proteins for organelle degradation. PINK1, a Ser/Thr kinase that accumulates only on impaired mitochondria, phosphorylates two authentic substrates, the ubiquitin-like domain of Parkin and ubiquitin. Our group and others have revealed that both the subcellular localization and ligase activity of Parkin are regulated through interactions with phosphorylated ubiquitin. Once PINK1 localizes on impaired mitochondria, PINK1-catalyzed phosphoubiquitin recruits and activates Parkin. Parkin then supplies a ubiquitin chain to PINK1 for phosphorylation. The amplified ubiquitin functions as a signal for the sequestration and degradation of the damaged mitochondria. Although a bewildering variety of Parkin substrates have been reported, the basis for Parkin substrate specificity remains poorly understood. Moreover, the mechanism underlying initial activation and translocation of Parkin onto mitochondria remains unclear, because the presence of ubiquitin on impaired mitochondria is thought to be a prerequisite for the initial PINK1 phosphorylation process. Here, we show that artificial mitochondria-targeted proteins are ubiquitylated by Parkin, suggesting that substrate specificity of Parkin is not determined by its amino acid sequence. Moreover, recruitment and activation of Parkin are delayed following depletion of the mitochondrial E3, MITOL/March5. We propose a model in which the initial step in Parkin recruitment and activation requires protein ubiquitylation by MITOL/March5 with subsequent PINK1-mediated phosphorylation. Because PINK1 and Parkin amplify the ubiquitin signal via a positive feedback loop, the low substrate specificity of Parkin might facilitate this amplification process.

## Introduction

Parkinson's disease (PD),^2^ one of the most prevalent neurodegenerative diseases, has a morbidity of 1% among the population aged 65 and over. Consequently, elucidation of its pathological and developmental mechanisms is of significant importance for an aging society. Linkage of respiratory chain deficiencies to sporadic PD patients were reported more than 20 years ago (1). Moreover, some pesticides such as rotenone and paraquat have been reported to cause PD-like phenotypes via inhibition of the mitochondrial electron transport system and accumulation of mitochondrial DNA mutations (2, 3). These results suggested that mitochondrial defects are relevant to PD. Although most PD cases are classified as sporadic, some arise from inherited familial Parkinsonism. To date, over 14 causal genes for familial Parkinsonism have been identified. PINK1 and Parkin are causal gene products for recessive familial Parkinsonism *PARK6* (Parkinson disease 6) and AR-JP (autosomal recessive juvenile parkinsonism, also called *PARK2*), respectively (4, 5). PINK1 and Parkin have crucial roles in the selective removal of impaired mitochondria (6–8). The mechanisms underlying activation and localization of Parkin, a ubiquitin ligating enzyme (E3), on damaged mitochondria have been elucidated in detail (6–8). However, in Parkin-catalyzed ubiquitylation, two questions remain to be elucidated: 1) how the substrate specificity of Parkin is determined and 2) how the initial ubiquitin that is a prerequisite for Parkin activation/translocation is conjugated to the outer mitochondrial membrane (OMM).

Given that many E3s have stringent substrate specificity, Parkin appears to be atypical. Parkin ubiquitylates a variety of OMM proteins on depolarized mitochondria. Indeed, hexokinase I (HKI), MitoNEET/CISD1, MFN (Mitofusin), Miro, Tom20/TOMM20, and VDAC (voltage-dependent anion-selective channel) have been reported as Parkin substrates by our group and others (9–19). Comprehensive ubiquitylome analyses following a decrease in mitochondrial membrane potential (ΔΨm) in Parkin-expressing cells accelerated identification of the Parkin substrate (20). Thorough identification of Parkin-and mitochondria depolarization–dependent diglycine (remnant of ubiquitylation) adduct peptides suggested that more than 2,000 proteins are ubiquitylated by Parkin in cells (21). Nevertheless, information about the structural and/or sequence determinants of a Parkin substrate remain limited.

Interestingly, during Parkin-catalyzed ubiquitylation, an interdependent relationship between Parkin and phosphorylated ubiquitin was observed. Although Parkin is recruited to depolarized mitochondria by a mitochondrial phosphoubiquitin chain, the localization of phosphorylated ubiquitin on damaged mitochondria depends on Parkin. Similarly, although latent Parkin is converted to an active E3 on depolarized mitochondria, the E3 activity of Parkin is a prerequisite for its localization on depolarized mitochondria (22–25). This interconnectedness suggests a positive feedback loop in which PINK1 phosphorylation of a mitochondrial ubiquitin chain recruits Parkin to the mitochondria to ubiquitylate OMM substrates with the resultant ubiquitin chain functioning as a PINK1 substrate again, thereby causing local accumulation of phosphorylated ubiquitin. Given that this loop triggers Parkin-mediated amplification of the phosphoubiquitin signal, the origin of the initial “seed” ubiquitin is of great interest.

In this study, we evaluated whether Parkin ubiquitylates mitochondria-targeted artificial proteins that are not intrinsic in mammalian cells. Furthermore, by using functional inhibition of mitochondrial E3s, we sought to identify the key E3 that catalyzes the initial “seed” ubiquitin on mitochondria. Our results suggest that Parkin requires no consensus sequence for substrate recognition. In addition, although phosphoubiquitylation of the substrate enhanced Parkin-catalyzed ubiquitylation, the results of *in vitro* reconstitution suggest that Parkin does not function as a phosphoubiquitin-recognizing E4. Rather, the phosphoubiquitin on the substrate accelerates recruitment of Parkin to the vicinity. We also revealed that ubiquitin routinely conjugated by MITOL/March5 has a significant role in the recruitment of Parkin to depolarized mitochondria.

## Results

### Ectopically mitochondria-localized proteins can be ubiquitylated by Parkin

**Figure 1.**
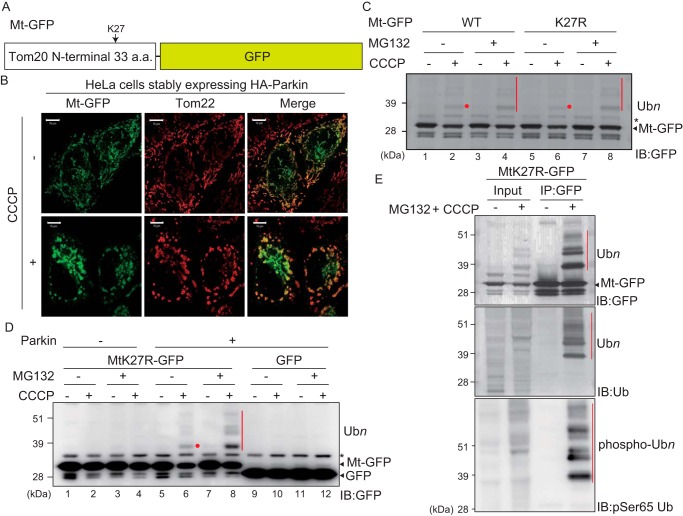
**Parkin-catalyzed ubiquitylation of mitochondria-localized GFP following CCCP treatment.** *A*, schematic representation of Mt-GFP. GFP was fused with the mitochondrial anchoring sequence of Tom20 (*i.e.* the N-terminal 33 amino acids (*a.a.*) of Tom20). *B*, mitochondrial localization of Mt-GFP. HeLa cells stably expressing HA–Parkin were transfected with Mt-GFP, treated with 15 μm CCCP for 3 h, and then subjected to immunocytochemistry. The Mt-GFP signal co-localized with the mitochondrial marker Tom22 ± CCCP. *Scale bars*, 10 μm. *C*, immunoblot-based (*IB*) detection of Mt-GFP ubiquitylation. HeLa cells stably expressing HA–Parkin were transfected with Mt-GFP or MtK27R–GFP, in which Lys-27 of the Tom20 mitochondrial anchoring sequence was mutated to Arg to prevent ubiquitin conjugation to the Tom20 moiety. The cells were treated with 15 μm CCCP ± 10 μm MG132 for 3 h and then immunoblotted with an anti-GFP antibody. The *red dots* and *bars* indicate ubiquitylated Mt-GFP, and the *black asterisk* indicates cross-reacting bands. *D*, mitochondrial GFP rather than cytosolic GFP is specifically ubiquitylated in a Parkin-dependent manner. HeLa cells stably expressing HA–Parkin transfected with MtK27R–GFP or GFP, and intact HeLa cells transfected with MtK27R–GFP were treated with 15 μm CCCP ± 10 μm MG132 for 3 h and then immunoblotted with an anti-GFP antibody. The *red dot* and *bar* indicate ubiquitylated Mt-GFP, and the *black asterisk* indicates a cross-reacting band. *E*, ubiquitylation of MtK27R–GFP involves ubiquitin and phosphoubiquitin. HeLa cells stably expressing HA–Parkin transfected with MtK27R–GFP were treated with 15 μm CCCP + 10 μm MG132 for 3 h and were then immunoprecipitated (*IP*) with an anti-GFP antibody. The total cell lysate (input) and immunoprecipitated products were immunoblotted with the indicated antibodies. The *red bars* indicate ubiquitylation (Ub*n*) or phosphoubiquitylation (phospho-Ub*n*) of MtK27R–GFP.

To investigate the range of Parkin substrate specificity, we artificially targeted GFP, which has numerous surface Lys residues (Protein Data Bank code 1GFL), to the mitochondrial outer membrane and examined Parkin-mediated ubiquitylation. If Parkin ubiquitylated the ectopically mitochondria-localized GFP after depolarization, it would suggest that Parkin substrate specificity is not determined by a specific amino acid sequence or a unique motif of genuine substrate proteins. The N-terminal 33 amino acids that correspond to the mitochondria-anchoring sequence of Tom20 were fused to GFP (referred to as Mt-GFP hereafter) (Fig. 1*A*) and then expressed in HeLa cells. The Mt-GFP signal overlapped well with the OMM protein Tom22/TOMM22 in immunocytochemistry (Fig. 1*B*), confirming mitochondrial localization. To determine whether Mt-GFP underwent ubiquitylation in response to mitochondrial depolarization, cells were treated with the uncoupling agent carbonyl cyanide *m*-chlorophenyl hydrazone (CCCP). A weak, higher-molecular mass band equivalent to ubiquitylation was observed following CCCP treatment (Fig. 1*C*, *lanes 1* and *2*). The signal was potentiated by addition of the proteasome inhibitor MG132 (Fig. 1*C*, *lanes 3* and *4*). A single lysine (Lys-27) in the mitochondria-anchoring sequence of Tom20, an authentic substrate of Parkin (26), is present in Mt-GFP. To exclude the possibility that this Tom20-derived residue is ubiquitylated by Parkin, we mutated the Lys to Arg (referred to as MtK27R–GFP hereafter). After CCCP treatment, MtK27R–GFP was likewise ubiquitylated (Fig. 1*C*, *lanes 6* and *8*), indicating that it is GFP rather than the Tom20 moiety that is ubiquitylated by Parkin.

To confirm the Parkin dependence, we examined MtK27R–GFP ubiquitylation in the absence or presence of Parkin. MtK27R–GFP was not ubiquitylated in intact HeLa cells lacking endogenous Parkin (27) but was ubiquitylated in HeLa cells expressing exogenous Parkin (Fig. 1*D*, compare *lane 2* with *lane 6* and *lane 4* with *lane 8*). Moreover, ubiquitylation of cytosolic GFP was not observed even after CCCP treatment in the presence of Parkin (Fig. 1*D*, *lanes 9–12*), indicating that OMM localization is a prerequisite for Parkin-mediated ubiquitylation of GFP.

To clarify that the higher molecular mass population of MtK27R–GFP was derived from ubiquitylation, cell lysates were immunoprecipitated with an anti-GFP antibody and then immunoblotted with anti-GFP, ubiquitin, and phospho(Ser-65)ubiquitin antibodies. These results clearly demonstrated that MtK27R–GFP was ubiquitylated (Fig. 1*E*, *lane 4*, *top* and *middle panels*) and that the MtK27R–GFP ubiquitin chains were partially phosphorylated (Fig. 1*E*, *bottom panel*). Taken together, these results indicate that Parkin can ubiquitylate exogenous GFP once it is targeted to mitochondria, suggesting that the key to Parkin recognition and ubiquitylation is mitochondrial localization rather than some consensus substrate sequence or motif.

**Figure 2.**
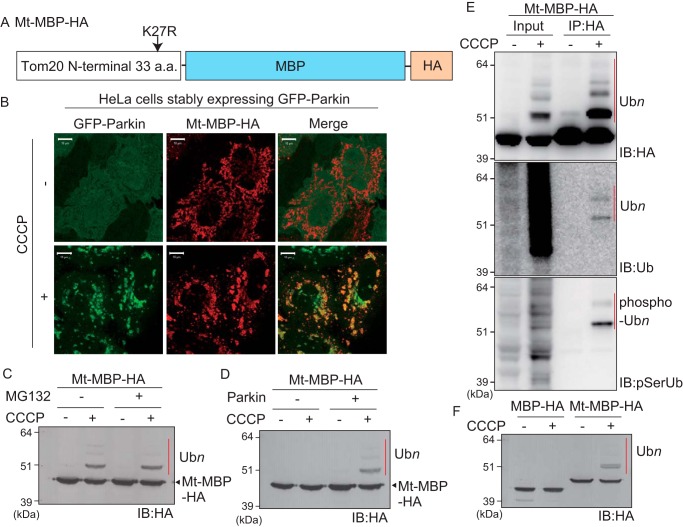
**Parkin-catalyzed ubiquitylation of mitochondria-localized MBP following CCCP treatment.** *A*, schematic representation of Mt–MBP–HA. *B*, mitochondrial localization of Mt–MBP–HA. HeLa cells stably expressing GFP–Parkin were transfected with Mt–MBP–HA, treated with 15 μm CCCP for 3 h, and then subjected to immunocytochemistry. Mt–MBP–HA localized to mitochondria with GFP–Parkin following a decrease in mitochondrial membrane potential. *Scale bars*, 10 μm. *C*, HeLa cells stably expressing GFP–Parkin transfected with Mt–MBP–HA were treated with 15 μm CCCP ± 10 μm MG132 for 3 h and then immunoblotted (*IB*) with an anti-HA antibody. The proteasomal inhibitor MG132 did not enhance ubiquitylation of Mt–MBP–HA. *D*, intact HeLa cells or HeLa cells stably expressing GFP–Parkin were transfected with Mt–MBP–HA and then immunoblotted with an anti-HA antibody. Mt–MBP–HA ubiquitylation depended on Parkin. *E*, ubiquitylation of Mt–MBP–HA involves ubiquitin and phosphoubiquitin. HeLa cells stably expressing GFP–Parkin transfected with Mt–MBP–HA were treated with 15 μm CCCP for 3 h and then immunoprecipitated (*IP*) with an anti-HA antibody. Total cell lysate (input) and the immunoprecipitated products were immunoblotted with the indicated antibodies. The *red bars* indicate ubiquitylation (Ub*n*) or phosphoubiquitylation (phospho-Ub*n*) of Mt–MBP–HA. *F*, Parkin ubiquitylates mitochondria-localized MBP-HA but not cytosolic MBP-HA. HeLa cells stably expressing GFP–Parkin transfected with Mt–MBP–HA or MBP-HA were treated with 15 μm CCCP for 3 h and were then immunoblotted with an anti-HA antibody. The *red bar* indicates Parkin-mediated ubiquitylation of Mt–MBP–HA. *a.a.*, amino acids.

To exclude the possibility that the GFP structural conformation resembles that of an endogenous Parkin substrate, we next tried to verify whether the mitochondria-localized prokaryotic protein maltose-binding protein (MBP) could likewise be recognized as a Parkin substrate and ubiquitylated. Similar to Mt-GFP (Fig. 1), the N-terminal 33 amino acids of Tom20 with the K27R mutation were fused to MBP. A Lys-free HA tag was added to the C terminus for detection with an anti-HA antibody (referred to as Mt–MBP–HA hereafter) (Fig. 2*A*). Like GFP, MBP has numerous surface Lys residues (Protein Data Bank code 1ANF). Immunocytochemical analysis showed mitochondrial localization of Mt–MBP–HA (Fig. 2*B*, *upper panel*) and that it co-localized with GFP–Parkin following a decrease in ΔΨm (Fig. 2*B*, *lower panel*). After CCCP treatment, Mt–MBP–HA underwent a higher molecular weight shift consistent with ubiquitylation irrespective of MG132 treatment (Fig. 2*C*). This shift was observed only in Parkin-expressing cells (Fig. 2*D*). Immunoprecipitation of Mt–MBP–HA with an anti-HA antibody followed by immunoblotting as before revealed conjugation of phosphorylated ubiquitin chains (Fig. 2*E*). When MBP-HA with and without the mitochondrial localization signal was expressed, only the mitochondria-targeted MBP was ubiquitylated following CCCP treatment, and cytosolic MBP did not undergo ubiquitylation (Fig. 2*F*). Collectively, exogenous GFP and MBP were ubiquitylated by Parkin upon mitochondrial localization.

### Ubiquitin fusion promotes Parkin-catalyzed ubiquitylation of a pseudomitochondrial substrate

**Figure 3.**
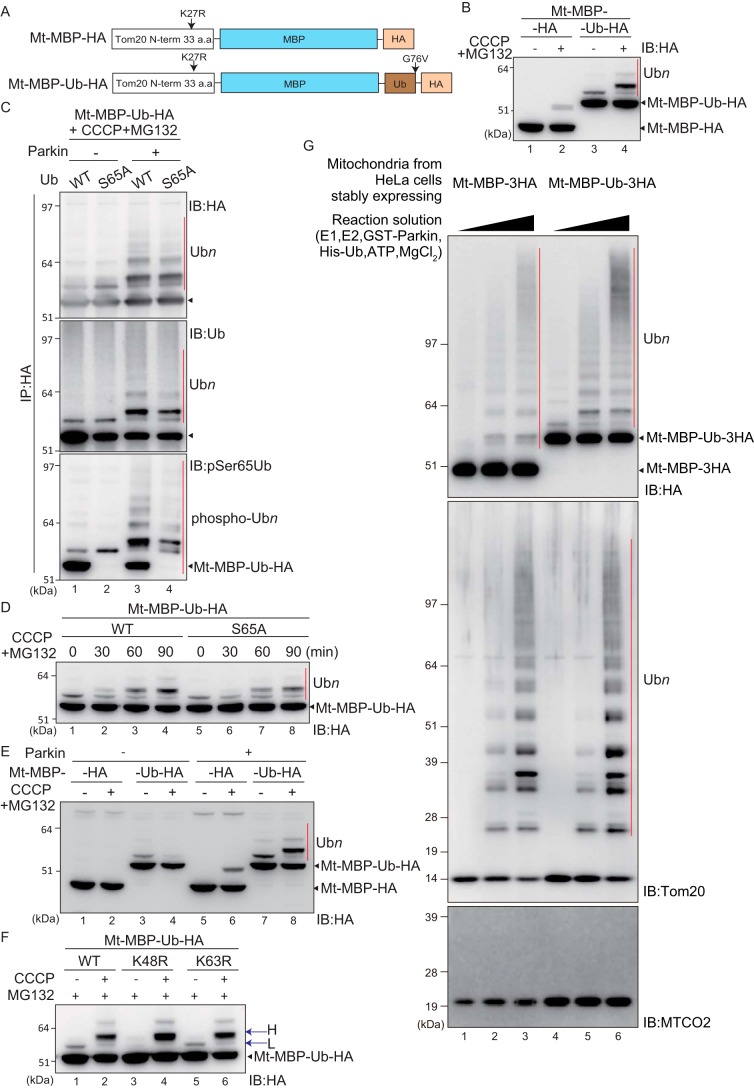
**Ubiquitin fusion accelerates Parkin-catalyzed ubiquitylation of mitochondria-localized MBP.** *A*, schematic representation of Mt–MBP–HA and Mt–MBP–Ub–HA. Gly-76 of the ubiquitin moiety was mutated to Val to avoid cleavage by DUB. *B*, HeLa cells stably expressing GFP–Parkin were transfected with Mt–MBP–HA or Mt–MBP–Ub–HA, treated with 15 μm CCCP + 10 μm MG132 for 3 h, and then immunoblotted (*IB*) with an anti-HA antibody. Mt–MBP–Ub–HA ubiquitylation (indicated by the *red bar*) was more efficient than that of Mt–MBP–HA. *C*, HeLa cells stably expressing GFP–Parkin were transfected with Mt–MBP–Ub–HA or Mt–MBP–Ub(S65A)–HA, treated with 15 μm CCCP + 10 μm MG132 for 3 h, and then immunoprecipitated (*IP*) with an anti-HA antibody. Immunoprecipitated products were immunoblotted with the indicated antibodies. The *red bars* indicate ubiquitylation (Ub*n*) or phosphoubiquitylation (phospho-Ub*n*) of Mt–MBP–Ub–HA and Mt–MBP–Ub(S65A)–HA. Phosphorylation of the ubiquitin moiety enhanced Parkin-catalyzed ubiquitylation of Mt–MBP–Ub–HA. *D*, HeLa cells stably expressing GFP–Parkin were transfected with Mt–MBP–Ub–HA or Mt–MBP–Ub(S65A)–HA, treated with 15 μm CCCP + 10 μm MG132 for the indicated times (min), and then immunoblotted with an anti-HA antibody. A phosphorylation-deficient S65A mutation in the ubiquitin moiety delayed Parkin-catalyzed ubiquitylation of Mt–MBP–Ub–HA. *E*, intact HeLa cells or HeLa cells stably expressing GFP–Parkin were transfected with Mt–MBP–HA or Mt–MBP–Ub–HA, treated with 15 μm CCCP + 10 μm MG132 for 3 h, and then immunoblotted with an anti-HA antibody. The *red bar* indicates ubiquitylation (Ub*n*) of Mt–MBP–Ub–HA. *F*, HeLa cells stably expressing GFP–Parkin were transfected with Mt–MBP–Ub–HA, Mt–MBP–Ub(K48R)–HA, or Mt-MBP-Ub(K63)-HA and then treated with 10 μm MG132 ± 15 μm CCCP for 3 h. Cell lysates were immunoblotted with an anti-HA antibody. *H* indicates higher molecular weight ubiquitylation that depends on both Parkin and CCCP; *L* indicates lower molecular weight Parkin-independent ubiquitylation. *G*, Parkin-mediated ubiquitylation of Mt–MBP–3HA and Mt–MBP–Ub–3HA reconstituted *in vitro*. Isolated mitochondria from CCCP-treated HeLa cells stably expressing Mt–MBP–3HA or Mt–MBP–Ub–3HA were mixed with recombinant ubiquitin, E1, E2 (UbcH7), and Parkin in the presence of ATP, MgCl_2_, and TCEP for 30 min *in vitro*. Ubiquitylation was analyzed by immunoblotting using anti-HA, anti-Tom20, and anti-MTCO2 antibodies. The *red bars* indicate ubiquitylation (Ub*n*) of Mt–MBP–3HA and Mt–MBP–Ub–3HA. *a.a.*, amino acids.

Although the data shown in Figs. 1 and 2 suggest that Parkin has low substrate specificity, an alternative interpretation could be that a common modification like ubiquitylation is a prerequisite for Parkin recognition and that Parkin assists in further ubiquitylation like E4, a ubiquitin chain-elongation factor. In this scenario, a common sequence is not expected among the substrates but ubiquitylation is expected to accelerate Parkin-mediated ubiquitylation/ubiquitin elongation. One ubiquitin was thus fused in-frame to Mt–MBP–HA (referred to as Mt–MBP–Ub–HA) (Fig. 3*A*), and then the fused ubiquitin (ubiquitin moiety) was examined for accelerated Parkin-catalyzed ubiquitylation. Mt–MBP–Ub–HA showed a band shift in both untreated and CCCP-treated conditions (Fig. 3*B*, *lanes 3* and *4*). Importantly, the Mt–MBP–HA band shift following CCCP treatment (derived from ubiquitylation as discussed below) was enhanced in Mt–MBP–Ub–HA (Fig. 3*B*, compare *lane 2* with *lane 4*), suggesting that the in-frame-fused ubiquitin accelerated further Parkin-catalyzed ubiquitylation.

To confirm that Mt–MBP–Ub–HA was ubiquitylated, samples were immunoprecipitated with an anti-HA antibody and then immunoblotted as before (“IB” in Fig. 3*C*). A shift in the Mt–MBP–Ub–HA band was detected with an anti-ubiquitin antibody (*lane 1*, *middle panel o*f Fig. 3*C*). Because Mt–MBP–Ub–HA with any kind of modification should be recognized by the anti-ubiquitin antibody, we introduced a phosphorylation-deficient S65A mutation into the ubiquitin moiety of Mt–MBP–Ub–HA (referred to as Mt–MBP–Ub(S65A)–HA) and used a custom anti-phosphoubiquitin antibody for immunoblotting (22). The anti-phosphoubiquitin antibody detected only the modified form of Mt–MBP–Ub(S65A)–HA (Fig. 3*C*, *lane 2*, *bottom panel*), whereas the anti-ubiquitin antibody detected both unmodified and modified forms of Mt–MBP–Ub(S65A)–HA (Fig. 3*C*, *lane 2*, *middle panel*). This result indicates that endogenous (phospho)ubiquitin is conjugated to Mt–MBP–Ub–HA even in the absence of Parkin. A similar experimental strategy revealed that endogenous (phospho)ubiquitin is also conjugated to Mt–MBP–Ub–HA in the presence of Parkin (Fig. 3*C*, *lanes 3* and *4*) (see “Discussion”). The positive immunoblotting signal generated by the anti-phosphoubiquitin antibody (Fig. 3*C*, *bottom panel*) revealed that the ubiquitin chains on Mt–MBP–Ub(S65A)–HA are phosphorylated.

Because the phosphorylation-deficient mutation (S65A) in the ubiquitin moiety seemed to attenuate ubiquitylation of Mt–MBP–Ub–HA by Parkin (Fig. 3*C*, compare *lane 3* with *lane 4*), more in-depth analysis was performed. When the effect of the S65A mutation within Mt–MBP–Ub–HA was examined following short intervals of CCCP treatment, the degree of ubiquitylation was reduced but not blocked (Fig. 3*D*). We next compared the ubiquitylation pattern of Mt–MBP–HA and Mt–MBP–Ub–HA in the absence/presence of Parkin and a decrease in ΔΨm (Fig. 3*E*). Ubiquitylation of Mt–MBP–HA depended on both Parkin and CCCP (Fig. 3*E*, *lane 6*) but was not observed in the absence of Parkin (Fig. 3*E*, *lane 2*) or CCCP (Fig. 3*E*, *lane 5*). In contrast, Mt–MBP–Ub–HA was ubiquitylated even under steady-state conditions irrespective of Parkin and ΔΨm (Fig. 3*E*, *lane 3*), but this constitutive ubiquitylation was considerably diminished after CCCP treatment (Fig. 3*E*, compare *lane 3* and *lane 4*). This process might be mediated by DUB (deubiquitylating enzyme) activity; however, we do not have any persuasive evidence for it, and thus there could be other interpretations.

Although the anti-phosphoubiquitin antibody revealed that Mt–MBP–Ub–HA was ubiquitylated both in the absence (Fig. 3*C*, *lanes 1* and *2*, *bottom panel*) and presence (Fig. 3*C*, *lanes 3* and *4*, *bottom panel*) of Parkin, the ubiquitylated Mt–MBP–Ub–HA band in untreated conditions (Fig. 3*E*, *lane 7*) was slightly lower than that under CCCP-treated conditions (Fig. 3*E*, *lane 8*). We thus speculated that the apparent molecular weight of the band might reflect different ubiquitin conjugation sites (*e.g.* either the MBP- or ubiquitin-moiety within Mt–MBP–Ub–HA). We thus mutated Lys-48 and Lys-63 within the ubiquitin moiety of Mt–MBP–Ub–HA to Arg (referred to as K48R or K63R) and then examined the ubiquitylation pattern (Fig. 3*F*). Ubiquitylation of Mt-MBP-Ub(K63R)-HA (Fig. 3*F*, *lanes 5* and *6*) was comparable with Mt–MBP–Ub–HA (Fig. 3*F*, *lanes 1* and *2*). In contrast, Mt–MBP–Ub(K48R)–HA had diminished ubiquitylation under untreated conditions (Fig. 3*F*, *lane 3*). Although ubiquitylation under steady-state conditions (marked with *L* in Fig. 3*F*) was affected by the K48R mutation, the higher ubiquitylation band that appear with Parkin and CCCP treatment (marked with *H* in Fig. 3*F*) was not reduced. These results suggest that Parkin preferentially ubiquitylates MBP to the ubiquitin moiety, and thus Parkin predominantly functions as an E3 rather than an E4.

To analyze Parkin-mediated ubiquitylation in greater detail, we performed an *in vitro* reconstitution assay. Isolated mitochondria from CCCP-treated HeLa cells stably expressing Mt–MBP–3HA or Mt–MBP–Ub–3HA were incubated with purified E1, E2 (His-UbcH7), and GST-rat Parkin in the presence of ATP, MgCl_2_, and TCEP *in vitro*. Reaction mixtures were then immunoblotted using anti-HA, anti-Tom20, and anti-MTCO2 (also known as cytochrome *c* oxidase subunit 2) antibodies. The outer mitochondrial membrane protein Tom20, an endogenous Parkin substrate, was efficiently ubiquitylated in proportion to the concentration of E1, E2, and Parkin (Fig. 3*G*, *middle panel*). In contrast, under the same experimental conditions, no ubiquitylation was observed for the mitochondrial matrix protein MTCO2 (Fig. 3*G*, *bottom panel*). Importantly, the *in vitro* ubiquitylation of both Mt–MBP–3HA and Mt–MBP–Ub–3HA by recombinant Parkin was indistinguishable. If Parkin is an E4, then (phospho)ubiquitylation of a substrate should function as an essential prerequisite signal for recognition, and Mt–MBP–Ub–3HA should be preferentially ubiquitylated by Parkin. However, as shown by our results, the Parkin-dependent ladder-like ubiquitylation pattern of Mt–MBP–3HA was nearly equivalent to that of Mt–MBP–Ub–3HA even in the reconstitution experiments, confirming that Parkin functions as an E3 rather than an E4 (Fig. 3*G*, *top panel*).

**Figure 4.**
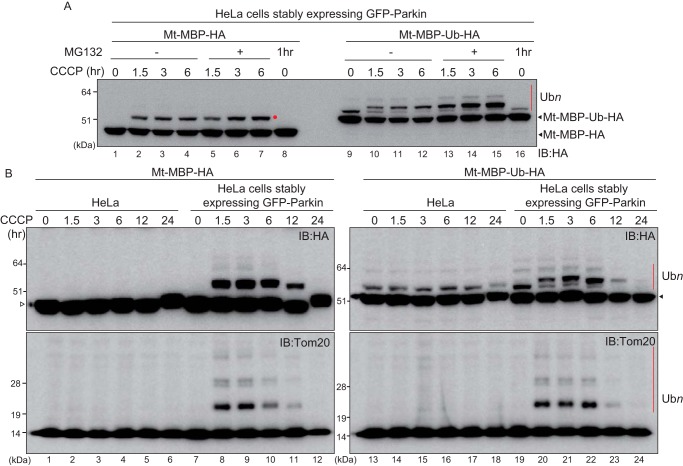
**Ubiquitylation of mitochondria-localized MBP and mitochondria-localized MBP-ubiquitin over time.** *A*, HeLa cells stably expressing GFP–Parkin were transfected with Mt–MBP–HA or Mt–MBP–Ub–HA and then treated with 15 μm CCCP ± 10 μm MG132 for the indicated times. Cell lysates were then immunoblotted (*IB*) with an anti-HA antibody. Unlike ubiquitylated Mt–MBP–HA (*lanes 1–7*), MG132 treatment promoted accumulation of ubiquitylated Mt–MBP–Ub–HA (*lanes 9–15*). The *red dot* and *red bar* indicate ubiquitylation of Mt–MBP–HA and Mt–MBP–Ub–HA, respectively. *B*, intact HeLa cells or HeLa cells stably expressing GFP–Parkin were transfected with Mt–MBP–HA or Mt–MBP–Ub–HA and then treated with 15 μm CCCP for the indicated times. Cell lysates were immunoblotted with an anti-HA antibody or an anti-Tom20 antibody. The *white triangle* by the *left panel* indicates Mt–MBP–HA, and the *black triangle* by the *right panel* indicates Mt–MBP–Ub–HA. The ubiquitylation patterns of both Mt–MBP–HA and Mt–MBP–Ub–HA were comparable with the ubiquitylation pattern of the endogenous substrate, Tom20. The *red bars* indicate ubiquitylation (Ub*n*) of Mt–MBP–Ub–HA and Tom20.

Next, to determine whether ubiquitylated Mt–MBP–Ub–HA is destined for proteasomal degradation, depolarized HeLa cells expressing GFP–Parkin and either Mt–MBP–HA or Mt–MBP–Ub–HA were treated with the proteasome inhibitor MG132. MG132 treatment did not affect accumulation or ubiquitylation of Mt–MBP–HA (Fig. 4*A*, *lanes 1–7*). Parkin- and CCCP-dependent ubiquitylation of Mt–MBP–Ub–HA, however, increased (Fig. 4*A*, *lanes 9–15*), indicating that Mt–MBP–Ub–HA ubiquitylated by Parkin is preferentially degraded by the proteasome. To follow the degradation process more precisely, cells transfected with Mt–MBP–HA or Mt–MBP–Ub–HA were exposed to CCCP along with the protein synthesis inhibitor cycloheximide (CHX). Mt–MBP–HA ubiquitylation depended on Parkin but did not undergo degradation until 6 h after CCCP and CHX treatment (Fig. S1, *lanes 1–8*). In contrast, Mt–MBP–Ub–HA ubiquitylated by Parkin was rapidly degraded (Fig. S1, *lanes 13–16*).

We also examined the ubiquitylation pattern of Mt–MBP–HA and Mt–MBP–Ub–HA after extended periods of CCCP treatment (up to 24 h). The same pattern of ubiquitylation as that shown in Fig. 3 was observed: Parkin-dependent ubiquitylation of Mt–MBP–HA (Fig. 4*B*, *lanes 7–12*), weak Parkin-independent monoubiquitylation of Mt–MBP–Ub–HA (Fig. 4*B*, *lanes 13–18*), and Parkin-dependent ladder formation of Mt–MBP–Ub–HA (Fig. 4*B*, *lanes 19–24*). The time-dependent ubiquitylation pattern of Mt-MBP was comparable with that of Tom20 (Fig. 4*B*, compare *upper* and *lower panels*, *lanes 7–12* and *19–24*).

Taken together, the results shown in Figs. 3 and 4 suggest that conjugation of the “seed” ubiquitin to a mitochondrial substrate enhances Parkin-mediated ubiquitylation. Because Parkin interacts with phosphorylated ubiquitin (6, 28), it is expected that a substrate previously ubiquitylated by another E3 is preferred by Parkin. Indeed, the in-frame fused ubiquitin in Mt–MBP–Ub–HA is phosphorylated (Fig. 3*C*), and accelerated Parkin-catalyzed ubiquitylation of Mt–MBP–Ub–HA is compared with Mt–MBP–HA (Fig. 3*B*). Introduction of the phosphorylation-deficient S65A mutation within the ubiquitin moiety, however, weakly abrogated Mt–MBP–Ub–HA ubiquitylation by Parkin (Fig. 3*D*). We thus speculated that ubiquitylation mediated by another mitochondrial E3 may contribute to Parkin translocation and subsequent ubiquitylation.

### MITOL knockdown weakens ubiquitylation of mitochondrial protein

Three mitochondria-localized ubiquitin ligases (E3s) have been reported to date: MuL-1, Huwe1, and MITOL (alternatively referred to as MARCH5). MuL-1 (mitochondria ubiquitin ligase 1), also known as MAPL (mitochondrial anchor protein ligase) and MULAN (mitochondrial ubiquitin ligase activator of NF-κB), is a RING finger protein that has been reported to function as both an E3 and SUMO ligase (29–32). Furthermore, Yun *et al.* (33) reported MuL-1 compensation of the PINK1/Parkin-mediated pathway in *Drosophila*. Huwe1 is a HECT-type E3 ligase that regulates apoptosis via interactions with phosphorylated mitofusin2 (34). MITOL is a RING finger E3 with four predicted transmembrane domains that has been reported to control mitochondrial dynamics by regulating mitochondrial fission/fusion factors such as Drp1, Fis1, and Mfn2 (35–40). MITOL has also been reported to control turnover of Mcl1/Bcl2-l-3 in cells (40).

**Figure 5.**
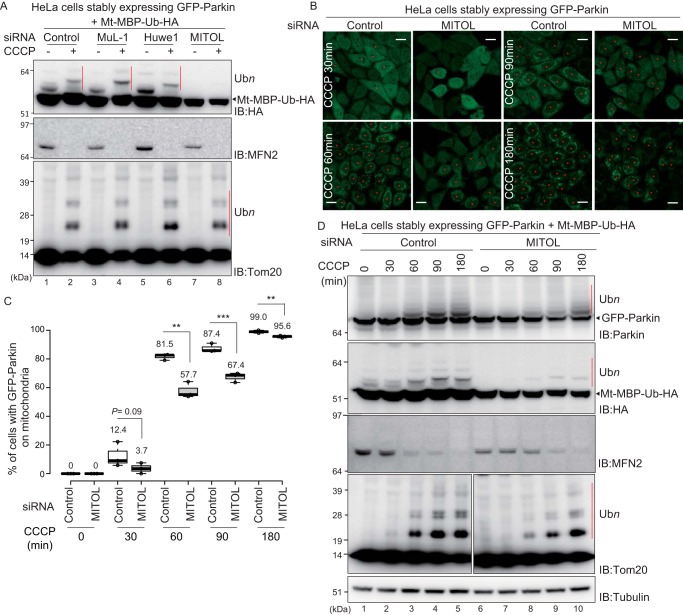
**Knockdown of *MITOL* attenuates Parkin recruitment to depolarized mitochondria and Parkin-catalyzed ubiquitylation.** *A*, HeLa cells stably expressing GFP–Parkin were simultaneously transfected with Mt–MBP–Ub–HA and siRNAs against three different mitochondrial E3s. The cells were incubated for 2 days and then treated with 15 μm CCCP for 3 h. Cell lysates were then immunoblotted (*IB*) with the indicated antibodies. The *red bars* indicate Parkin-catalyzed ubiquitylation (Ub*n*) of Mt–MBP–Ub–HA and Tom20. *MITOL* knockdown attenuated the ubiquitylation of Mt–MBP–Ub–HA. *B*, HeLa cells stably expressing GFP–Parkin were transfected with a control siRNA or siRNA for *MITOL*, followed by CCCP treatment (15 μm) for the indicated times, and then the subcellular localization of GFP–Parkin was observed. The *red asterisks* indicate cells in which GFP–Parkin was recruited to mitochondria. *Scale bars*, 20 μm. *C*, statistical analysis of the subcellular localization of GFP–Parkin following 15 μm CCCP treatment for the indicated times in cells treated with control or *MITOL* siRNA. The percentage of cells with the indicated GFP–Parkin localization was calculated using > 100 cells. The *numbers* in the box-and-whisker plot are mean values across three independent experiments. Statistical significance was calculated using a one-tailed Student's *t* test. **, *p* < 0.01; ***, *p* < 0.001. *D*, HeLa cells stably expressing GFP–Parkin were simultaneously transfected with Mt–MBP–Ub–HA and control siRNA or siRNA for *MITOL*. The cells were incubated for 2 days and then treated with 15 μm CCCP for the indicated times. The cell lysates were then immunoblotted with the indicated antibodies. The *red bars* indicate autoubiquitylation of GFP–Parkin or Parkin-catalyzed ubiquitylation of Mt–MBP–Ub–HA and Tom20.

To examine the contribution of these E3s in Parkin-mediated ubiquitylation of mitochondrial proteins, each was knocked down using siRNAs in HeLa cells stably expressing GFP–Parkin. After transfection with Mt–MBP–Ub–HA, cells were treated with CCCP, and their cell lysates were immunoblotted (Fig. 5*A*). Knockdown of *MuL-1* and *Huwe1* had no effect on the ubiquitylation pattern of MFN2, Tom20 (endogenous Parkin substrate), or Mt–MBP–Ub–HA, whereas ubiquitylation of Mt–MBP–Ub–HA was reduced following *MITOL* siRNA transfection (Fig. 5*A*, *lane 8*).

After confirming that *MITOL* was successfully knocked down (Fig. S2), we next compared GFP–Parkin recruitment in *MITOL* knockdown cells and control siRNA-treated cells. Recruitment of GFP–Parkin was significantly delayed following *MITOL* knockdown (Fig. 5, *B* and *C*), suggesting that MITOL positively regulates the recruitment of Parkin early in mitophagy. To verify the effect of MITOL on mitochondrial ubiquitylation more precisely, the ubiquitylation of mitochondrial proteins was examined after short intervals of CCCP treatment. Consistent with the previous results (Fig. 5*A*), the autoubiquitylation of GFP–Parkin, the ubiquitylation of the artificial substrate Mt–MBP–Ub–HA, and the ubiquitylation or degradation of the endogenous substrates (Tom20 and MFN2) were delayed by *MITOL* knockdown (Fig. 5*D*). The effects of *MITOL* knockdown on Tom20 ubiquitylation and MFN2 degradation were more prominent than those shown in Fig. 5*A* because the extended CCCP exposure time concealed the minor differences in the endogenous substrates.

**Figure 6.**
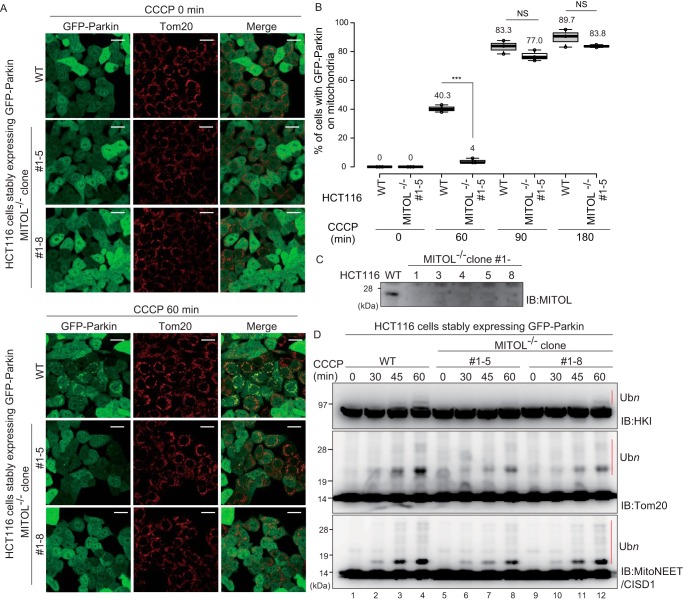
**MITOL knockout attenuates Parkin recruitment to depolarized mitochondria and Parkin-catalyzed ubiquitylation.** *A*, *MITOL* knockout HCT116 cells were generated using the CRISPR/Cas9 system. Intact HCT116 cells and *MITOL* knockout HCT116 cells stably expressing GFP–Parkin were treated with 15 μm CCCP for 60 min, and then the subcellular localization of GFP–Parkin was observed. *Scale bars*, 20 μm. *B*, statistical analysis of GFP–Parkin mitochondrial localization following 15 μm CCCP treatment for the indicated times in WT or *MITOL* knockout HCT116 cells. The percentage of cells with Parkin-positive mitochondria were determined using >100 cells. The *numbers* in the box-and-whisker plot are mean values across three independent experiments. Statistical significance was calculated using a one-tailed Student's *t* test. ***, *p* < 0.001. *NS*, not significant. *C*, MITOL depletion in *MITOL* knockout HCT116 cells was verified by immunoblotting (*IB*) with an anti-MITOL antibody. *D*, WT or *MITOL* knockout HCT116 cells stably expressing GFP–Parkin were treated with 15 μm CCCP for the indicated times and then immunoblotted with the indicated antibodies. The *red bars* indicate Parkin-catalyzed ubiquitylation (Ub*n*) of the endogenous substrates HKI, Tom20, and MitoNEET/CISD1.

To ensure the absence of off-target effects in the *MITOL* siRNA assays, *MITOL* knockout HCT116 cells were generated using the CRISPR-Cas9 system. Using these cells, we assessed the rate of ubiquitylation of OMM-localized proteins and Parkin recruitment to damaged mitochondria. Similar to the *MITOL* siRNA assays, significant differences in the recruitment of Parkin to the damaged mitochondria was observed between WT and *MITOL* knockout HCT116 cells after 60 min CCCP treatment (Fig. 6, *A* and *B*). However, the defect in Parkin translocation to damaged mitochondria in the *MITOL* knockout HCT116 cells was not detected when CCCP treatment exceeded 90 min. We speculate that the function of MITOL in Parkin recruitment is limited to initiating Parkin-mediated mitophagy and that amplification of ubiquitin chains by Parkin on damaged mitochondria after extensive CCCP treatment abrogates the initial negative effect of MITOL depletion. Moreover, the *MITOL* knockout slightly weakened ubiquitylation of the endogenous Parkin substrates HKI, MitoNEET/CISD1, and Tom20 during a short time course of mitophagy after CCCP treatment (Fig. 6*D*).

**Figure 7.**
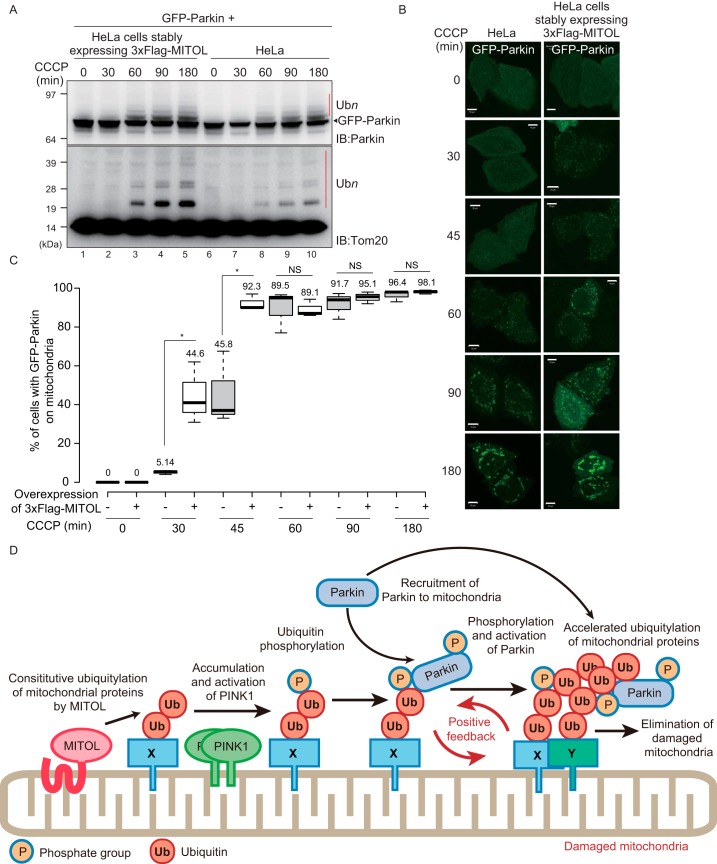
**Overexpression of MITOL enhances Parkin recruitment to depolarized mitochondria and Parkin-catalyzed ubiquitylation.** *A*, intact HeLa cells or HeLa cells stably expressing 3×FLAG–MITOL were transfected with GFP–Parkin, treated with 15 μm CCCP for the indicated times, and then immunoblotted (*IB*) with anti-Parkin and anti-Tom20 antibodies. The *red bars* (Ub*n*) indicate autoubiquitylation of GFP–Parkin or Parkin-catalyzed ubiquitylation of Tom20. *B*, subcellular localization of transiently transfected GFP–Parkin in intact HeLa cells or HeLa cells stably expressing 3×FLAG–MITOL following 15 μm CCCP treatment for the indicated times. *Scale bars*, 10 μm. *C*, statistical analysis of the subcellular localization of GFP–Parkin following 15 μm CCCP treatment for the indicated times in intact HeLa cells or HeLa cells stably expressing 3×FLAG–MITOL. The percentage of cells with the mitochondria GFP–Parkin localization was calculated using >100 cells. *Numbers* in the box-and-whisker plot are mean values across three independent experiments. Statistical significance was calculated using a one-tailed Student's *t* test. *, *p* < 0.05. *B* and *C* show that overexpression of MITOL accelerates the recruitment of GFP–Parkin onto impaired mitochondria. *D*, schematic model. At steady-state conditions, MITOL ubiquitylates the endogenous mitochondrial substrate. When the mitochondrial membrane potential decreases, this MITOL-catalyzed constitutive ubiquitylation is used as a “seed” ubiquitin for subsequent PINK1/Parkin-dependent ubiquitylation, thereby generating a positive feedback cycle that amplifies ubiquitylation. Because Parkin lacks substrate specificity, the amplified ubiquitylation of proteins on damaged mitochondria can proceed efficiently. *X*, MITOL substrate; *Y*, any type of mitochondrial protein as Parkin lacks substrate specificity; *NS*, not significant.

Finally, as a complementary experiment, we determined whether MITOL overexpression accelerated Parkin-mediated ubiquitylation of OMM proteins. HeLa cells stably expressing 3×FLAG–MITOL or WT HeLa cells were transfected with GFP–Parkin, followed by CCCP treatment, and cell lysates were immunoblotted with anti-Parkin and anti-Tom20 antibodies (Fig. 7*A*). Autoubiquitylation of GFP–Parkin and ubiquitylation of Tom20 following CCCP treatment were potentiated by overexpression of 3×FLAG–MITOL as compared with WT HeLa cells. We next evaluated the rate of GFP–Parkin recruitment in 3×FLAG–MITOL-overexpressing HeLa cells. The translocation of GFP–Parkin onto impaired mitochondria in 3×FLAG–MITOL–overexpressed HeLa cells was more rapid than in WT HeLa cells, in particular at 30–45 min after CCCP treatment (Fig. 7, *B* and *C*). These results indicate that excess MITOL accelerated Parkin recruitment and subsequent ubiquitylation of OMM proteins. We next examined whether MITOL E3 activity is essential for accelerating Parkin recruitment to damaged mitochondria. HeLa cells stably expressing GFP–Parkin and WT MITOL or an E3 inactive C65S/C68S (CS) mutant MITOL were treated with CCCP, and then the number of cells with Parkin-positive mitochondria was determined over time (Fig. S3). At 30–45 min CCCP treatment, cells expressing the MITOL (CS) mutant had fewer Parkin-positive mitochondria than cells expressing WT MITOL (Fig. S3), indicating that the acceleration of Parkin recruitment to depolarized mitochondria depends on the E3 activity of MITOL. However, at the later time points (>60 min), the difference between cells expressing WT MITOL and the CS MITOL mutant was negligible (Fig. S3). This pattern is reminiscent of Parkin translocation in both MITOL-expressing and non–MITOL-expressing cells (Fig. 6*B*).

Taken together, the results shown in Figs. 6 and 7 suggest that MITOL ubiquitylates a genuine OMM substrate such as Mcl1/Bcl2-L-3 (40) under steady-state conditions and that MITOL-mediated ubiquitin-conjugated OMM proteins might be phosphorylated by PINK1 to function as a “seed” for recruitment and activation of Parkin. This then induces the positive feedback loop of ubiquitylation on depolarized mitochondria, as depicted in Fig. 7*D*.

## Discussion

Our group and others have revealed that translocation of Parkin to mitochondria depends on phosphorylated ubiquitin chains on depolarized mitochondria that form via a positive feedback loop among PINK1, Parkin, and phosphorylated ubiquitin (28). Moreover, it has also been reported that once targeted to depolarized mitochondria, Parkin ubiquitylates a diverse number of proteins on damaged mitochondria (20, 21). The former observation raised questions regarding how the initial ubiquitin in the positive feedback cycle was attached to mitochondria, and the latter results were confounding as such broad substrate specificity contrasted with typical E3 activity. To address these issues, we first examined the substrate specificity of Parkin using artificial substrates and then investigated the involvement of other mitochondrial E3s in efficient PINK1/Parkin-mediated mitochondrial ubiquitylation.

From the mitochondrial E3 knockdown results, we found that a reduction of MITOL delayed Parkin recruitment to depolarized mitochondria, and decreased the efficiency of Parkin-mediated mitochondrial ubiquitylation. In MITOL-depleted cells, mitochondrial recruitment and autoubiquitylation of GFP–Parkin, degradation of MFN2, and the ubiquitylation of both an exogenous substrate (Mt–MBP–Ub–HA) and endogenous substrates (Tom20, HKI, and MitoNEET/CISD1) were reduced (Figs. 5 and 6). Although the effect of MITOL was more pronounced with synthetic substrates (*e.g.* Mt–MBP–Ub–HA) than native substrates, a similar effect, albeit less robust, was observed with endogenous proteins (*e.g.* Tom20), suggesting that our hypothesis is applicable to genuine substrates as well. The overexpression of MITOL accelerated the mitochondrial localization and autoubiquitylation of GFP–Parkin, as well as the ubiquitylation of the endogenous substrate Tom20 (Fig. 7), an effect that depends on the E3 activity of MITOL (Fig. S3). While conducting the experiments, we realized that the effect of MITOL dysfunction on Parkin had a more drastic effect on mitochondrial translocation than substrate ubiquitylation. Indeed, differences in ubiquitylation of Tom20 and MitoNEET between control and MITOL-depleted cells were somewhat subtle (Figs. 5*D* and 6*D*), whereas the inhibitory effect on Parkin recruitment to impaired mitochondria was pronounced in MITOL-depleted cells (Figs. 5, *B* and *C*, and 6, *A* and *B*). According to the current prevailing hypothesis, phosphorylated ubiquitin on mitochondria cause structural changes in Parkin that facilitate subsequent phosphorylation of the ubiquitin-like (Ubl) domain and conversion to the active form (41, 42). Under this scenario, it seems unexplainable that MITOL-catalyzed ubiquitylation is more effective for Parkin recruitment than for Parkin activation. Although the phosphorylated ubiquitin chain on mitochondria that acts as a Parkin receptor is attenuated in MITOL-depleted cells, the levels of cytosolic phosphorylated ubiquitin might be unchanged, and thus Parkin could still be converted to the active form.

Regardless, we propose that MITOL introduces the initial “seed” ubiquitylation that promotes rapid Parkin recruitment at the onset of mitophagy, as depicted in Fig. 7*D*. GFP and MBP are nongenuine proteins for mammalian cells that have numerous surface-exposed Lys residues but share no common motifs. Consequently, the ubiquitylation of mitochondria-targeted GFP and MBP by Parkin (Figs. 1 and 2) strongly suggests that Parkin does not have a consensus substrate recognition motif/sequence. This conclusion is consistent with several previous reports. In 2012, Lazarou *et al.* (43) reported that when PINK1 and Parkin were ectopically targeted to the peroxisome, they induced pexophagy. The result seems strange from the viewpoint that peroxisome should lack a genuine Parkin substrate. However, if we assume that Parkin has no substrate specificity and thus can ubiquitylate peroxisomal proteins, there is no inconsistency even if Parkin induces pexophagy. Moreover, low substrate specificity of Parkin is compatible with the fact that an enormous number of mitochondrial proteins are ubiquitylated by Parkin as reported previously (21).

Nevertheless, even if no consensus sequence is identified in the various substrates, it is possible that the substrates have a common modification or state that is required for Parkin recognition. For example, Parkin has been reported to ubiquitylate misfolded proteins, and reactive oxygen species from damaged mitochondria have been reported to promote Parkin-mediated ubiquitylation (44). It is thus possible that reactive oxygen species–induced misfolding is a common prerequisite signal for Parkin-mediated ubiquitylation of various mitochondrial proteins. However, we found that a constitutively active PINK1 mutant promotes ubiquitylation on normal (energized) mitochondria (45). Although misfolding-induced Parkin-catalyzed ubiquitylation cannot be ruled out, it is likely not a prerequisite for Parkin-catalyzed ubiquitylation because it is doubtful that active PINK1 induces the misfolding of various proteins on normal mitochondria.

Alternatively, the following hypothesis is attractive in that proteins ubiquitylated by various E3s are phosphorylated by PINK1 and that “phosphoubiquitylation” is the signal recognized by Parkin, because Parkin binds phosphorylated ubiquitin (46–48). We thus examined whether artificial phosphoubiquitylation accelerated further Parkin-catalyzed ubiquitylation (Fig. 3). Ubiquitin was fused in-frame to the artificial mitochondrial substrate Mt–MBP–HA (referred to as Mt–MBP–Ub–HA). When immunoprecipitated Mt–MBP–Ub–HA products were blotted with an anti-phosphoubiquitin antibody, it was clear that the ubiquitin portion of Mt–MBP–Ub–HA was phosphorylated (Fig. 3*C*). Parkin-dependent ubiquitylation of mitochondria-targeted MBP is promoted by in-frame fusion of ubiquitin (Fig. 3*B*) and is decreased by a phosphorylation-deficient mutation (S65A) in the ubiquitin moiety (Fig. 3*D*). However, the promotional effect by (phospho)ubiquitin fusion is not drastic. This raises the question of how we should consider the straightforward hypothesis that phosphoubiquitylation itself is a motif for Parkin-mediated recognition and ubiquitylation (*i.e.* whether Parkin functions as phosphoubiquitin-recognizing E4). If a substrate is initially phosphoubiquitylated beforehand and the modification functions as an essential “prerequisite signal” for recognition by Parkin, subsequent Parkin-dependent ubiquitylation should proceed from the diubiquitylated position. However, based on our experimental data, the substrates exhibited a Parkin-dependent ladder-like ubiquitylation pattern that started from the monoubiquitylated position in both cells (Fig. 6*D*) and a fully reconstituted *in vitro* system (Fig. 3*G*). Therefore, phosphoubiquitylation is not an essential “prerequiste signal” for recognition as a substrate by Parkin but is rather a facilitator for Parkin-mediated ubiquitylation. Accelerated Parkin translocation to the mitochondria by the presence of phosphoubiquitylated proteins (including Mt–MBP–Ub–HA) might reflect enhanced ubiquitylation of mitochondrial proteins (Fig. 3*D*).

Conclusively, it is reasonable to think that Parkin has basically no substrate selectivity at the sequence and modification levels, except when substrates are phosphoubiquitylated, Parkin ubiquitylates them more efficiently. We can surmise that the low substrate specificity of Parkin is advantageous for quickly ubiquitylating multiple proteins on impaired mitochondria and to advance the positive feedback cycle. Instead of low substrate specificity, the recruitment and activation of Parkin are strictly regulated at multiple points (6) to ensure pinpoint removal of damaged mitochondria in cells.

## Experimental procedures

### Plasmids and antibodies

To prepare the Mt-GFP and Mt-HA plasmids, the DNA fragment coding the N-terminal 33 amino acids of Tom20 was inserted into the BamHI site of the pEGFP-N1 (Clontech) and pcDNA3/C-terminal HA vectors, respectively. *MBP* lacking the signal sequence was subcloned into the EcoRI site of Mt-HA to generate the Mt–MBP–HA plasmid. To construct a plasmid for stable expression of 3×FLAG–MITOL, the MITOL coding sequence was PCR-amplified from a HeLa cDNA library and inserted into the BamHI/EcoRI sites of the pMXs-puro/N-terminal 3×FLAG vector. For Mt–MBP–Ub–HA plasmid construction, the ubiquitin G76V coding sequence was inserted into the EcoRV site of the Mt–MBP–HA plasmid. The Tom20 K27R and ubiquitin S65A, K48R, and K63R mutations were introduced by primer-based PCR mutagenesis. For the *in vitro* ubiquitylation assay, pT7-7 plasmids encoding His_6_-human ubiquitin and His_6_-UbcH7, and pGEX6P1 plasmids encoding GST-human E1-His_8_ and GST-rat Parkin were used.

The following antibodies were used for immunoblotting: anti-GFP (ab6556, Abcam), anti-HA (TANA2, MBL), anti-tubulin (YL1/2, Abcam), anti-Parkin (PRK8, Sigma), anti-MFN2 (ab56889, Abcam), anti-Tom20 (FL145, Santa Cruz), anti-hexokinase I (C35-C4, Cell Signaling), anti-MitoNEET/CISD1 (16006-1-AP, ProteinTech Group, Inc.), anti-MITOL (kind gift from Dr. Yanagi), anti-ubiquitin (P4D1, Santa Cruz), and anti–Ser-65 phosphorylated ubiquitin (custom made) (22). The following antibodies were used for immunocytochemistry: anti-HA (TANA2, MBL), anti-Tom22 (1C9-2, Sigma), anti-Tom20 (FL145, Santa Cruz), anti-MTCO2 (ab110258, Abcam), and anti–Ser-65 phosphorylated ubiquitin (custom made) (22).

### Reconstituted ubiquitylation assay

After 15 μm CCCP treatment for 6 h, HeLa cells expressing Mt–MBP–3HA or Mt–MBP–Ub–3HA were suspended in cell-free assay buffer (0.25 m sucrose and 50 mm Tris-HCl, pH 7.5) supplemented with a protease inhibitor mixture minus EDTA (Roche). The cells were disrupted by passaging 30 times through a 25-gauge needle, and cell homogenates were centrifuged at 1,000 × *g* for 7 min at 4 °C to obtain a postnuclear supernatant. Mitochondria were pelleted by two rounds of centrifugation at 10,000 × *g* for 10 min at 4 °C. To perform the cell-free ubiquitylation assay, recombinant His_6_-human ubiquitin (maximum, 30 μm), GST-E1-His_8_ (maximum, 0.154 μm), His_6_-UbcH7 (max. 6 μm), and GST-rat Parkin (maximum, 3 μm) were incubated with isolated mitochondria in the reaction buffer (50 mm Tris-HCl, pH 7.5, 5 mm ATP, 10 mm MgCl_2_, 125 mm NaCl, 1 mm TCEP, 220 mm mannitol, and 70 mm sucrose) at 32 °C for 30 min. Recombinant ubiquitin, E1, E2, and Parkin were prepared as previously reported (47). The samples were washed with cell-free assay buffer twice, solubilized with SDS-PAGE sample buffer, and then immunoblotted.

### Cells and transfections

HeLa cells were cultured at 37 °C with 5% CO_2_ in Dulbecco's modified Eagle's medium (Gibco) containing 1× nonessential amino acids (Gibco), 1× sodium pyruvate (Gibco), and 10% fetal bovine serum. HCT116 cells were cultured in McCoy's 5A medium (Gibco) supplemented with 1× nonessential amino acids (Gibco), 10% fetal bovine serum, and 1× GlutaMAX (Gibco). To generate HeLa cells stably expressing HA–Parkin or GFP–Parkin, HeLa cells transiently expressing mCAT1 (murine cationic amino acid transporter-1) were infected with recombinant retroviruses harboring HA–Parkin or GFP–Parkin. Recombinant retrovirus was produced using PLAT-E cells as described previously (49). HeLa cells stably expressing 3×FLAG–MITOL and HCT116 cells stably expressing GFP–Parkin were established by recombinant retrovirus infection. Vector particles were made in HEK293T cells by co-transfection with Gag–Pol, vesicular stomatitis virus G glycoprotein, and the retrovirus plasmids (50). After 12 h of transfection, the culture medium was replaced with fresh medium. The cells were further cultivated for 24 h. Collected viral supernatants were then used to infect HeLa cells or HCT116 cells with 8 μg/ml Polybrene (Sigma). Plasmid transfections were performed using FuGENE 6 (Promega) and polyethyleneinime (Polyscience) according to the manufacturer's instructions. To depolarize mitochondria, the cells were treated with 15 μm CCCP (Wako) for 3 h unless otherwise specified. To inhibit proteasome activity, 10 μm MG132 (Sigma) was used, and 50 μg/ml CHX (Sigma) was used to inhibit protein translation.

### RNAi

Nontargeting control siRNA (siGENOME control pool 1, nontargeting 1, catalogue no. D-001206-13-20) was purchased from Thermo Scientific. siRNA oligonucleotides for MuL-1, Huwe1, and MITOL were purchased from Qiagen. The target sequences are as follows: MuL-1 1, 5′-CCGCGCCTTGCCAGAGCCCAA-3′; Huwe1 1, 5′-AAGCAGCTTATGGAGATTAAA-3′; and MITOL 1, 5′-CAGGAATAATGGTCGGCTCTA-3′. siRNAs were transfected into cells using Lipofectaine RNAiMAX (Invitrogen) according to the manufacturer's instructions. After 6 h of transfection, the medium was replaced with fresh medium, and the cells were grown for another 42 h.

### Establishment of MITOL KO cell line by CRISPR/Cas9-based genome editing

*MITOL*^−/−^HCT116 cells were generated by CRISPR/Cas9-based genome editing with an antibiotics-selection strategy. The gRNA target sequence for a region of exon 2 in the *MITOL* gene (5′-cca ggc ctg tct aca acg ctg gg-3′) was selected using an online CRISPR design tool (CRISPR direct). Two DNA oligonucleotides, hMITOL–ex2-1–CRISPR-F (5′-TGT ATG AGA CCA Ccc agg cct gtc tac aac gct-3′) and hMITOL–ex2-1–CRISPR-R (5′-AAA Cag cgt tgt aga cag gcc tgg GTG GTC TCA-3′) were annealed and introduced into a linearized EF1–hspCas9–H1–gRNA vector (Cas9 SmartNuclease^TM^, System Biosciences, LLC) according to the manufacturer's protocol. The DNA fragment was verified by sequencing. Neomycin- and hygromycin-resistant marker plasmids were constructed as follows. The neomycin-resistant gene (*NeoR*), including loxP sites along with the appropriate promoter and terminator, was PCR-amplified from pMK286 (51) using BamHI–NeoR-F (5′-ggc cGG ATC Cct aat taa cta gAT AAC TTC GTA TAA TGT ATG CTA TAC GAA GTT ATc tga ggc gga aag aa-3′) and NeoR–BamHI-R (5′-GGC Cgg atc cAT AAC TTC GTA TAG CAT ACA TTA TAC GAA GTT Ata acg acc caa cac cg-3′) primers. The hygromycin-resistant gene (*HygroR*), including loxP sites with appropriate promoter and terminator, was PCR-amplified from pMK287 (51) using BamHI–HygroR-F (5′-ggc cGG ATC Cct aat taa cta gAT AAC TTC GTA TAA T-3′) and HygroR–BamHI-R (5′-ggc cGG ATC Cta gtg aac ctc ttc g-3′) primers. The amplified *NeoR* and *HygroR* fragments were digested with BamHI and ligated into the corresponding site of pBluescriptII SK(−) to make pBSK/NeoR and pBSK/HygroR, respectively. The 247 bp of the 5′ and 3′ homology arms of the *MITOL* exon 2 region, which lacks the gRNA target sequence but has a BamHI site in the middle (total 500 bp), was synthesized and cloned into a pUC57-Amp vector (GENEWIZ) to make pUC57–Amp/MITOL–ex2–donor. *NeoR* and *HygroR* resistant markers extracted by BamHI digestion from pBSK/NeoR and pBSK/HygroR were then inserted into the BamHI site of pUC57–Amp/MITOL–ex2–donor. The resultant *NeoR* and *HygroR* donor plasmids containing the *MITOL* exon 2 homology arm were transfected into HCT116 cells with the gRNA plasmid using FuGENE 6. The cells were grown in McCoy's 5A medium containing 700 μg/ml G418 (G8168, Sigma) and 100 μg/ml hygromycin B (10687-10, Invitrogen). The single colonies were isolated and genomic DNA was extracted using Quick-gDNA MicroPrep (ZYMO Research). Insertion of the *NeoR* and *HygroR* markers into the exon 2 region of *MITOL* gene was confirmed by PCR, and MITOL protein knockout was confirmed by immunoblotting with an anti-MITOL antibody.

### Immunoblotting

HeLa cells were solubilized with TNE-N^+^ buffer (20 mm Tris-HCl, pH 8.0, 150 mm NaCl, 1 mm EDTA, 1% Nonidet P-40, and protease-inhibitor mixture complete EDTA-free; Roche) in the presence of 1 mm *N*-ethylmaleimide. After removing insoluble debris by centrifugation, the supernatant was collected to obtain total cell lysates. The total protein concentration of the lysates was determined using a BCA protein assay kit (Pierce). SDS-PAGE sample buffer was added to lysates, and samples were boiled at 98 °C for 5 min. Proteins were separated on 4–12% Bis-Tris SDS-PAGE gels (NuPAGE, Invitrogen) in MOPS buffer. Proteins were transferred to polyvinylidene difluoride membranes, blocked with 5% skim milk/TBST, and incubated with primary antibodies. To detect phosphorylated ubiquitin, polyvinylidene difluoride membranes were blocked with Blocking One-P (Nacalai Tesque) and incubated with an anti–Ser-65 phosphorylated ubiquitin antibody/MaxBlot (MBL). The membranes were then incubated with horseradish peroxidase–conjugated goat anti-mouse, goat anti-rabbit, and donkey anti-rat antibodies (antibodies 315-035-048, 111-035-144, and 712-035-153; Jackson ImmunoResearch Inc.) as secondary antibodies. The images were obtained on an ImageQuant LAS 4000 (GE Healthcare).

### Immunoprecipitation

HeLa cells grown in a 10-cm dish were solubilized with TNE-N^+^ buffer. After centrifugation, the supernatant was incubated with anti–HA-agarose (A2095, Sigma) or GFP-trap (GFP–Trap A, ChromoTek) for 3 h at 4 °C. The agarose was washed three times with TNE-N^+^ buffer, and proteins were extracted by adding SDS-PAGE sample buffer.

### Immunocytochemistry

HeLa cells were fixed with 4% paraformaldehyde (Wako), permeabilized with 50 μg/ml digitonin (Wako), and incubated with primary antibodies followed by 1:2000 secondary antibodies (Alexa Fluor 488- or 568-conjugated goat anti-mouse or anti-rabbit IgG antibody; Invitrogen). Microscopy images were captured on a laser-scanning microscope (LSM710 or LSM780; Carl Zeiss), and image brightness was adjusted with Photoshop software (Adobe).

## Author contributions

F. K. resources; F. K. data curation; F. K. formal analysis; F. K., K. Y., H. K., K. T., and N. M. funding acquisition; F. K. investigation; F. K. visualization; F. K. and H. K. methodology; F. K. and N. M. writing-original draft; K. Y. and N. M. conceptualization; K. Y. and N. M. validation; K. Y., H. K., K. T., and N. M. writing-review and editing; K. T. and N. M. supervision; K. T. and N. M. project administration.

## Supplementary Material

Supporting Information

## Footnotes

2 The abbreviations used are:

PD: Parkinson's disease
OMM: outer mitochondrial membrane
HKI: hexokinase I
CCCP: carbonyl cyanide *m*-chlorophenyl hydrazone
MBP: maltose-binding protein
TCEP: tris(2-carboxyethyl)phosphine
CHX: cycloheximide.
