# Supplementary material for: Parkin recruitment to impaired mitochondria for nonselective ubiquitylation is facilitated by MITOL
Source: J Biol Chem. 2019 May 20;294(26):10300–14. doi: 10.1074/jbc.RA118.006302 (PMC6664184; doi:10.1074/jbc.RA118.006302)
Supplement: Supporting Information [file supp_294_26_10300__index.html]

Parkin recruitment to impaired mitochondria for nonselective ubiquitylation is facilitated by MITOL — MITOL assists Parkin in mitochondrial localization — Parkin recruitment to impaired mitochondria for nonselective ubiquitylation is facilitated by MITOL — MITOL assists Parkin in mitochondrial localization — Supporting Information 

# Parkin recruitment to impaired mitochondria for nonselective ubiquitylation is facilitated by MITOL

## Supporting Information

- Supporting Information - Supporting Information. Koyano et al.
