# Supplementary material for: Parkin recruitment to impaired mitochondria for nonselective ubiquitylation is facilitated by MITOL
Source: J Biol Chem. 2019 May 20;294(26):10300–14. doi: 10.1074/jbc.RA118.006302 (PMC6664184; doi:10.1074/jbc.RA118.006302)
Supplement: Supporting Information [file supp_RA118.006302_141369_1_supp_315239_pqyymv.pdf]

Supporting Information

Parkin recruitment to impaired mitochondria for nonselective ubiquitylation is facilitated by MITOL

Fumika Koyano[1], Koji Yamano[1], Hidetaka Kosako[2], Keiji Tanaka[3], and *Noriyuki Matsuda[1]

[1]Ubiquitin Project, Tokyo Metropolitan Institute of Medical Science, 2-1-6 Kamikitazawa, Setagaya, Tokyo 156-8506, Japan, [2]Division of Cell Signaling, Fujii Memorial Institute of Medical Sciences, Tokushima University, 3-18-15 Kuramoto-cho, Tokushima 770-8503, Japan, [3]Laboratory of Protein Metabolism, Tokyo Metropolitan Institute of Medical Science, 2-1-6 Kamikitazawa, Setagaya, Tokyo 156-8506, Japan

*To whom correspondence should be addressed: matsuda-nr@igakuken.or.jp

Running title: MITOL assists Parkin in mitochondrial localization

Keywords: Parkin, Parkinson’ s disease, mitophagy, ubiquitin, ubiquitin ligase

Table of content

Figure S1: The ubiquitylation and degradation pattern of Mt-MBP-HA and Mt-MBP-Ub-HA after CCCP and cycloheximide (CHX).

Figure S2: Endogenous MITOL was reduced in *MITOL*-knock down cells.

Figure S3: Statistical analysis of the subcellular localization of GFP-Parkin in HeLa cells stably expressing 3xFlag-MITOL WT or an E3-inactive C65S/C68S (CS) mutant following CCCP treatment.

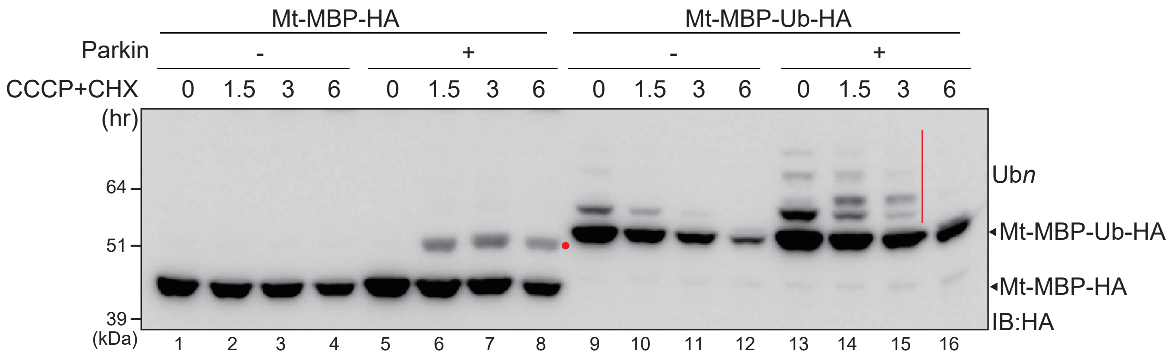

Figure S1.

Intact HeLa cells or HeLa cells stably expressing GFP-Parkin were transfected with Mt-MBP-HA or Mt-MBP-Ub-HA. They were then treated with 15 μM CCCP + 50 μg/ml cycloheximide (CHX) for the indicated times (hr), and then immunoblotted with an anti-HA antibody. The red dot and red bar indicate ubiquitylation of Mt-MBP-HA and Mt-MBP-Ub-HA, respectively.

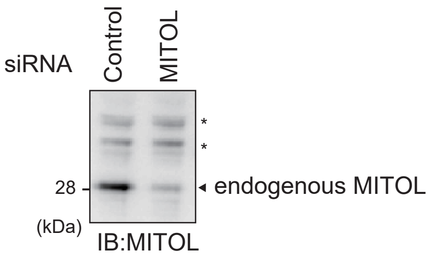

Figure S2.

HeLa cell lysates treated with control or *MITOL* siRNA were immunoblotted with an anti-MITOL antibody. Endogenous MITOL was reduced in *MITOL*-knock down cells. The asterisks indicate cross-reacting bands.

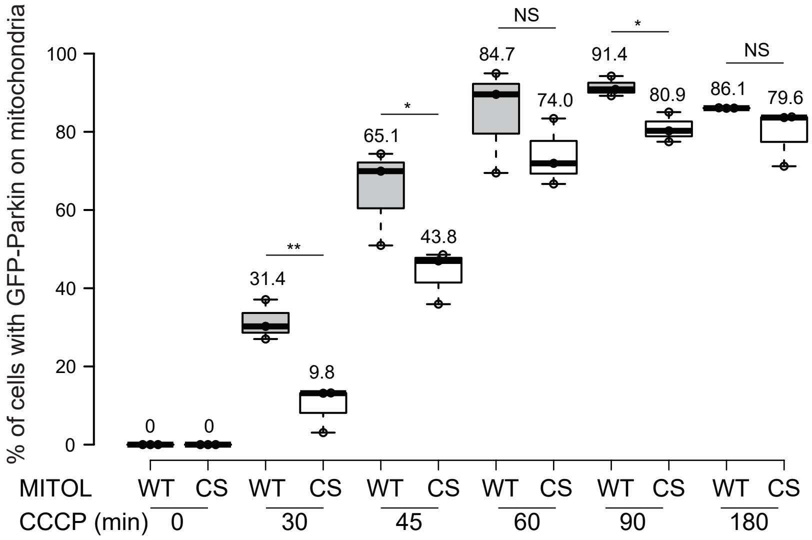

Figure S3.

Statistical analysis of the subcellular localization of GFP-Parkin in HeLa cells stably expressing 3xFlag-MITOL WT or an E3-inactive C65S/C68S (CS) mutant following 15 μM CCCP treatment for the indicated times (min).

The percentages of cells with Parkin-positive mitochondria were calculated using > 100 cells.

Numbers in the box-and-whisker plot are mean values across three independent experiments.

Statistical significance was calculated using a one-tailed Welch's *t*-test. *, $P<0.05$; **, $P<0.01$. NS, Not Significant.
